# A comprehensive review of obstructive sleep apnea

**DOI:** 10.5935/1984-0063.20200056

**Published:** 2021

**Authors:** Anna Abbasi, Sushilkumar Satish Gupta, Nitin Sabharwal, Vineet Meghrajani, Shaurya Sharma, Stephan Kamholz, Yizhak Kupfer

**Affiliations:** 1 Maimonides Medical Center, Medicine - Brooklyn - NY - United States.; 2 Maimonides Medical Center, Pulmonary and Critical Care Medicine - Brooklyn - NY - United States.

**Keywords:** Sleep Apnea, Obstructive, Airway Obstruction, Sleep Apnea Syndromes

## Abstract

Obstructive sleep apnea (OSA) is a complex disorder characterized by collapse of the upper airway during sleep. Downstream effects involve the cardiovascular, pulmonary, and neurocognitive systems. OSA is more prevalent in men than women. Clinical symptoms suggest the diagnosis of OSA but none is pathognomonic of the condition. With rising awareness of OSA and the increasing prevalence of obesity, OSA is increasingly recognized as a major contributor to cardiovascular morbidity including systemic and pulmonary arterial hypertension, heart failure, acute coronary syndromes, atrial fibrillation, and other arrhythmias. Pulmonary manifestations include the development of chronic thromboembolic disease, which can then lead to chronic thromboembolic pulmonary hypertension (CTEPH). Neurocognitive morbidities include stroke and neurobehavioral disorders. Screening for OSA includes the use of symptom questionnaires and the diagnosis is confirmed by polysomnography. Management primarily includes the use of continuous positive airway pressure (CPAP) or bi-level positive airway pressure (BiPAP) devices during sleep. Alternate options such as mandibular devices and surgical procedures are considered for certain patient populations.

## INTRODUCTION

Obstructive sleep apnea (OSA) is a disorder caused by upper airway obstruction (which can be partial or complete) during sleep^1^. The change in airway muscle tone during sleep leads to collapse of the upper airways (predominantly during the inspiratory phase of breathing), which leads to intermittent episodes of hypopnea and/or apnea^2^^,^^3^. During these episodes the arterial oxygen saturation falls, which can lead to autonomic dysregulation^3^. These acute changes result over time in chronic conditions that affect the cardiovascular, pulmonary, and neurocognitive systems^2^^,^^3^. In 1956, Bickelmann et al.^4^ described obesity hypoventilation syndrome (OHS) in a report regarding an obese business executive who presented to the hospital complaining of excessive daytime sleepiness. These investigators attributed the name “Pickwickian syndrome” to the condition based on the description of a similar fictional character in Charles Dickens’ first novel (1836-37) “The posthumous papers of the Pickwick Club”^4^. Following that, numerous descriptions of the obesity hypoventilation syndrome, central sleep apnea and obstructive sleep apnea were published under the rubric of sleep- disordered breathing (SDB).

Currently, obstructive sleep apnea (OSA) is the most prevalent, clinically significant SDB, and it is known to be associated with numerous diseases including hypertension, atrial fibrillation, heart failure, cerebrovascular accidents, pulmonary hypertension and others^2^^,^^5^^,^^6^. The goal of this article is to compile and therefore understand the impact OSA has on other organ systems.

### Prevalence and risk factors

OSA is more prevalent in men than women^1^^,^^2^. Benjafield et al.^7^ performed an extensive review to gauge the worldwide prevalence of this disease, which consisted of reliable prevalence data from 16 countries including Brazil, Germany, Spain, China, Switzerland, and USA. The worldwide prevalence of OSA was extrapolated from this data and showed that about 1 billion people aged 30-65 years are affected by OSA, 425 million of those deemed to have moderate to severe OSA^7^. The prevalence of SDB among 30-49-year-old men in North America is 10% compared to 3% among women in the same age bracket, and 17% among 50-70-year-old men, compared to 9% among women in the same age bracket^3^.

Numerous risk factors are associated with OSA that can be detected on physical examination.

- Obesity and high body mass index are the strongest risk factors predisposing OSA. There is a linear correlation between OSA and obesity^1^^,^^6^;
- Neck circumference greater than 17 inches (43cm) in men and 15 inches (38cm) in women^8^;
- Male gender^1^;
- Age more than 50 years^1^^,^^2^;
- Other risk factors include menopause, neuropathy or myopathy that may affect the upper airway muscles (particularly the genioglossus muscle), craniofacial anatomical structure (particularly in the Asian population), family history, smoking, and nasal congestion^2^^,^^5^^,^^8^.
- Patients with acromegaly may have OSA due to macroglossia and they develop central sleep apnea due to altered respiratory control^9^.

### Clinical symptoms

Clinical symptoms play a key role in identifying patients with OSA but none is pathognomonic of the disease.

Patients usually complain of fatigue, excessive daytime sleepiness, snoring, drooling, nocturnal gasping or choking, headaches and/or falling asleep while driving^10^. Patients with OSA are more likely to be involved in motor vehicle collisions^10^.

The purpose of this article is to review the prevalence, risk factors, clinical presentation, effects of OSA on different organ systems, diagnostic criteria, and to discuss current approaches to the management of patients with OSA.

### OSA & cardiovascular disease

OSA is well recognized as a major contributor of cardiovascular disease. Previous studies have not definitively identified the direct link between OSA and cardiovascular disease, since patients with OSA often have other risk factors for cardiovascular disease such as hypertension (HTN), obesity, diabetes, and smoking. Theoretical explanations of the association of OSA and cardiovascular disease include the observations that OSA produces a chronic inflammatory state, which leads to increased atherosclerotic changes in the blood vessels of the patient.

**Figure 1:**
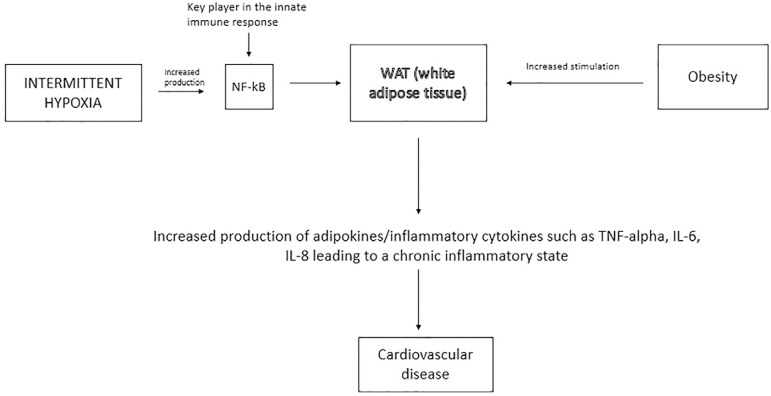
Pathogenesis of cardiovascular disease secondary to OSA.

Similar to obesity, which is also considered a low-grade inflammatory state, OSA has been shown to stimulate the white adipose tissue (WAT) leading to the production of inflammatory mediators^11^. OSA leads to sleep fragmentation, intermittent hypoxemia and in some instances, recurrent hypercapnia, which in turn, stimulate increased sympathetic activity, increased systemic inflammation, and increased oxidative stress. The end result of these changes is endothelial dysfunction and metabolic dysfunction which accounts for the increase in cardiovascular disease. Among the aforementioned factors, intermittent hypoxemia has been shown to play a critical role by promoting increased production of inflammatory markers, as depicted in Figure 1 ^11^^-^^14^.

### OSA & systemic hypertension

Evidence has been growing steadily for systemic arterial hypertension (HTN) and OSA as cardiovascular disease risk factors. In 1980, Lugaresi et al.^15^ associated systemic hypertension with snoring in the general population. In 1985, Fletcher et al.^16^ evaluated forty-six middle aged/older men with essential hypertension and thirty-four normotensive, age and weight matched controls for undiagnosed sleep apnea. Thirteen in the study group and three in the control group were found to have undiagnosed sleep apnea. Seven men with hypertension and sleep apnea were treated with protriptyline and one underwent uvulopalatopharyngoplasty (UPPP). A reduction in mean blood pressure (BP) was observed (149/95mmHg to 139/90mmHg) accompanied by a significant decrease in the apnea-hypopnea index (AHI) by 77%. The investigators concluded that OSA could be either the cause or a contributor to systemic arterial hypertension^16^.

Due to the prevalence of obesity as a confounding factor among studied patient populations, the confirmation of the association between OSA and HTN has been challenging^17^^,^^18^. However, more recently association of nocturnal OSA and daytime hypertension has been demonstrated, even after the adjustment for body mass index^19^^-^^21^. A prospective longitudinal cohort study with 1889 participants followed for 12.2 years (median) years identified an independent association of OSA and HTN from the confounders including age and obesity. The study demonstrated an increased hazard ratio for incident HTN in patients with OSA compared to the control subjects. Further follow up revealed a dose-response relationship between the severity of OSA and the cumulative incidence of HTN^22^.

**Figure 2:**
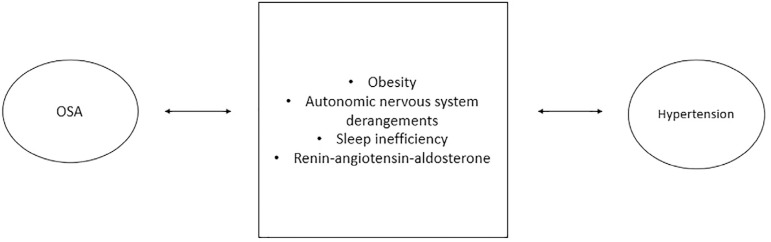
Association of OSA and hypertension.

The pathophysiology of the association between OSA and HTN is multifactorial. The potential causative factors are summarized in Figure 2.

Obesity has been identified as an independent risk factor both for OSA and HTN^22^. Adipose tissue deposition in the oropharyngeal around the upper airway contributes to apnea.

Downstream effects of OSA include physical inactivity, poor dietary habits, insulin resistance, hyperleptinemia, and systemic inflammation. The vicious cycle between obesity and apnea exacerbates HTN^23^^-^^26^. The nocturnal dipping pattern of BP is a normal physiological phenomenon that is affected by OSA^27^. Intermittent hypoxia and hypercapnia cause autonomic derangements which lead to nighttime increases in BP, and increased catecholamine levels which persist during daytime, worsening HTN^28^. OSA is well known to cause sleep inefficiency, which in itself has been shown to be correlated with several cardiovascular risk factors such as non-dipping of nocturnal BP, endothelial dysfunction, arterial stiffness, and increased sympathetic activity. The renin-angiotensin system (RAS) is known to be activated by obesity and more recently, it is known to be activated by OSA. The presence of OSA has a cumulative effect on obesity-induced activation of RAS, thereby worsening HTN^29^.

Phillips et al performed a randomized controlled trial to compare the change in cardiovascular and neurobehavioral outcomes among patients using CPAP and mandibular advancement devices (MAD). They found that although CPAP was more effective at reducing AHI, patient compliance was better with MADs. They also found that neither treatment improved blood pressure^30^. However, the meta-analysis by Montesi et al.^31^ demonstrated a significant reduction in the systolic and diastolic blood pressure of patients when treated with CPAP for their OSA. Reduced nighttime BP and sympathetic traffic can be achieved with effective OSA treatment resulting in more successful BP control^31^.

### OSA & heart failure

Sleep-disordered breathing (SDB), (central sleep apnea [CSA] and OSA), was found to be more prevalent in patients with heart failure (HF)^32^. However, OSA often remains as an undiagnosed risk and contributing factor for heart failure. Paulino et al.^33^ demonstrated that the prevalence of sleep breathing disorder was 81% (n=256) in 316 patients, 30% of whom were classified as CSA and 70% as OSA. Among this cohort of patients, both CSA and OSA existed together due to certain pathophysiological changes brought on by heart failure leading to SDB.

**Figure 3:**
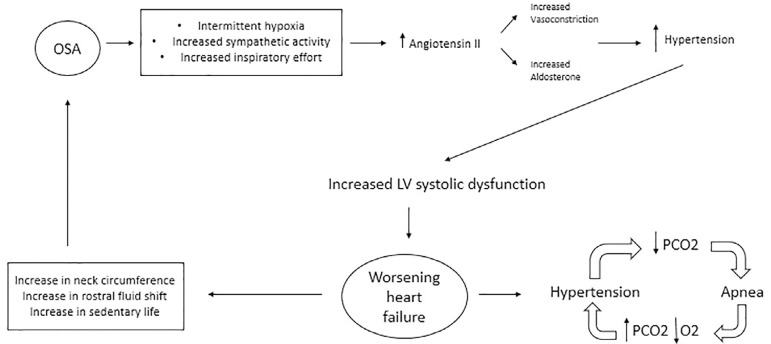
Association of OSA and heart failure.

The interactions of the heart failure and OSA are illustrated in Figure 3, OSA can lead to intermittent hypoxemia, which activates inflammatory pathways and promotes oxidative stress. Increased inspiratory effort against the high resistance of the upper airway leads to increased arousals and, combined with intermittent hypoxia, leads to sympathetic activation^11^. In addition, the high negative intrathoracic pressure exerted during inspiration through a narrowed or occluded upper airway may increase pulmonary capillary fluid efflux contributing to interstitial edema.

Increased sympathetic activity leads to increased angiotensin II release which is a potent vasoconstrictor promoting aldosterone production via the adrenal cortex. Aldosterone increases water and salt reabsorption leading to increased intravascular volume, worsening HTN and heart failure^34^. Chronic untreated OSA can lead to persistently elevated BP with absent nighttime dipping of BP, contributing to left ventricular systolic dysfunction^35^.

There are varied mechanisms by which heart failure may worsen OSA and induce apnea. Fluid overload leading to upper airway (UA) narrowing and upper airway edema are important mechanisms leading to worsening OSA^36^. In systolic heart failure patients, nighttime rostral fluid shift from the legs is a contributing pathophysiological mechanism causing worsening of OSA in HF^37^. Overnight rostral leg fluid displacement and an increase in neck circumference have been shown to significantly worsen OSA and CSA. There is a positive correlation with the volume of lower extremity extravascular fluid volume^37^. The brain stem respiratory centers in the medulla and pons receive input from peripheral arteries and the respiratory system. The effectors for the ventilatory center are the respiratory muscles. Heart failure leads to hypoxemia and increased afferent activity from the juxta capillary receptors (due to pulmonary capillary engorgement) which in turn drives the ventilatory center leading to hyperventilation^38^. Hyperventilation leads to decreased PaCO2 levels, resulting in hypopnea and/or apnea.

Also, increased circulation time leads to slower feedback from the chemoreceptors in the peripheral arteries. Pulmonary vascular congestion and edema lead to lower alveolar PAO2 and PACO2 reserve adds to ventilatory instability^39^.

### OSA and acute coronary syndrome

OSA is prevalent in patients with preexisting cardiovascular diseases. The plausible factors contributing to the development and maintenance of cardiovascular impairment in patients with OSA include intermittent hypoxemia, development of acidosis, increase in blood pressure and sympathetic vasoconstriction^11^.

The prospective sleep heart health study (SHHS)^40^ was done to establish the association between OSA and incident coronary artery disease and heart failure. For the purposes of data collection and analysis regarding incident coronary disease, the first occurrence of myocardial infarction, coronary heart disease (CHD), death, or coronary revascularization procedures were included. The rate of incident CHD was 20.1 events per 1000 person-years of follow-up in men and 8.7 events per 1,000 person-years in women. Event rates increased with severity of OSA in men. When these models were adjusted for age, race, BMI, and smoking status, there was a significant association of apnea/hypopnea index (AHI) with incident CHD in men but not in women. However, this association was not statistically significant after accounting for diabetes mellitus and lipid measures, adjustment for systolic and diastolic blood pressure and anti-hypertensive medication use also diminished the significance of the association.

Other prospective studies have shown an increased association between OSA and CHD. Shah et al.^41^ assessed whether OSA increased the risk of cardiovascular events. The outcomes studied included MI, coronary artery revascularization procedures and death from cardiovascular causes. These investigators found that patients with OSA had an increased risk of these outcomes, despite controlling for other cardiovascular risk factors including diabetes, hypertension, hyperlipidemia, tobacco, and alcohol use. A systematic review by Porto et al.^42^ supported an association between OSA and MI, which was greater in men.

Another observational study compared cardiovascular outcomes (fatal and non-fatal) in men with OSA who were being treated with CPAP versus untreated men with OSA^43^. Fatal myocardial infarction (MI) and stroke, non-fatal MI, non-fatal stroke, coronary artery bypass surgery, and coronary angiography were the study parameters. Severe OSA significantly increased the risk of fatal and non-fatal cardiovascular outcomes. The study also demonstrated that CPAP treatment in patients with severe OSA reduced the aforementioned adverse outcomes and those patients with mild and moderate OSA, did not exhibit increased risk of these outcomes. Buchner et al.^44^ reported that OSA treatment with CPAP had a benefit for patients with all severities of OSA and resulted reduced adverse cardiovascular outcomes. Interestingly the SAVE (sleep apnea cardiovascular endpoints) study^45^, a randomized control trial conducted to assess the effects of CPAP on major cardiovascular events, showed that CPAP use did not prevent cardiovascular events in patients with moderate to severe sleep apnea. It did reduce snoring and daytime sleepiness and improved health-related quality of life and mood. Interestingly the observational study conducted by Anandam et al.^46^ showed that CPAPs and MADs may have similar effectiveness in reducing cardiovascular mortality.

### OSA and atrial fibrillation

Atrial fibrillation (AF) is the most common arrhythmia linked with OSA. The prevalence of AF in patients with known OSA is 5%^47^. OSA may trigger the onset of and contributes to its persistence^48^. However, OSA is more common and less frequently detected in patients with AF^47^^,^^49^. The severity of OSA correlated with a higher incidence of AF and may also be a predictor of AF recurrence after cardioversion and/or ablation procedures^50^^,^^51^. There is limited success of antiarrhythmic therapy in patients with severe OSA^52^. Patients with OSA are more likely to develop AF post coronary artery by-pass graft surgery (CABG)^53^. OSA is associated with an increased incidence of AF in patients with heart failure^54^, coronary artery disease (CAD), and hypertrophic cardiomyopathy (HCM)^55^.

### Pathophysiology of developing AF in patients with OSA

Mechanisms linked to the development of AF in patients with OSA include the hemodynamic changes occurring during the apneic episodes. Hypoxemia and hypercapnia during episodes of hypopnea/apnea leads to tachycardia and hypertension. These changes are accompanied by increased myocardial oxygen demand, despite the restricted supply of oxygen during these episodes. This leads to myocardial injury and fibrosis, which promote the development of AF^11^^,^^47^. Episodes of hypopnea/apnea and post apneic reoxygenation also lead to oxidative stress, which contributes to the remodeling of the myocardium^47^. OSA is associated with higher levels of CRP and IL-6^56^, however the use of CPAP therapy has been shown to decrease these inflammatory markers in patients with OSA^56^.

OSA is also associated with atrial enlargement, conduction abnormalities, and prolonged sinus node recovery time^57^. This structural and electrical remodeling contributes to the development of AF.

Negative intrathoracic pressure during apneic episodes may be associated with the development of AF. The Mueller maneuver was performed on healthy adults to simulate the changes in the upper airway, which occur during OSA^58^ and found that the negative intrathoracic pressure led to a decrease in the left atrial volume, increase in left ventricular systolic function and an increase in the ventricular afterload. These dynamic changes can be implicated in the development of AF. Repetitive cycles of negative intrathoracic pressure also lead to atrial stretch, which contributes to the enlargement of the chambers and development of AF^48^. Negative intratracheal pressure causes shortening of the atrial effective refractory period through vagal stimulation, which also predisposes to the development of AF^59^.

### Management of AF in OSA

OSA is considered a modifiable risk factor for AF^47^. Current evidence suggests that continuous positive airway pressure is the standard treatment for OSA. Shah et al.^60^ showed that CPAP had a beneficial effect on left ventricular remodeling. Use of CPAP is associated with effective lowering of blood pressure, decreased atrial size and ventricular mass, and a lower risk for AF recurrence after ablation^61^. Recurrence of AF after cardioversion is also less frequent in patients being treated with CPAP compared to patients not being treated with^62^. Bayir et al.^63^ showed that after 6 months of therapy with CPAP in patients with OSA, there was an improvement in the interatrial, left intra-atrial and right intra-atrial electromechanical delays when compared to pretreatment measurements and possibly a decreased risk for OSA related AF^63^. CPAP can also help reverse left atrial volumetric abnormalities in as little as 12 weeks and improve left atrial remodeling over a period of 24 weeks^64^. Furthermore, it reduces the risk of progression from paroxysmal AF to persistent AF^65^. Drug refractory AF is often treated with catheter ablation (or a convergent procedure); however, screening patients with atrial fibrillation for OSA prior to catheter ablation may be beneficial, and could possibly obviate the need for the procedure^66^.

Renal nerve denervation (RND) is an emerging modality that may be of benefit^66^. Despite the failure of RND in managing drug resistant hypertension, there are new experimental studies suggesting that it may control arrhythmias caused by hyperactivity of the sympathetic nervous system^67^^,^^68^. Linz et al.^68^ studied the effects of RND on anesthetized pigs and determined that RND reduced AF caused by negative intratracheal pressure and reduced shortening of the atrial effective refractory period, in contrast to administration of atenolol which did achieve the same results^68^. They also reported that RND prevented post apneic elevation in blood pressure, decreased plasma renin activity and aldosterone levels^68^.

### OSA and other arrhythmias

OSA is associated other arrhythmias including ventricular tachycardia, premature ventricular contractions, ventricular fibrillation, sinus bradycardia and sick sinus syndrome (SSS)^69^^,^^70^.

OSA is also associated with bradycardia, long pauses and sick sinus syndrome. Simantirakis et al.^71^ conducted a study on 23 patients with an established diagnosis of OSA to evaluate their arrhythmias and to determine the effect of CPAP therapy on those arrhythmias. These investigators noted that these patients had multiple episodes of bradycardia and long pauses during sleep. Their study also showed that treatment with CPAP reduced these episodes. In another report, the prevalence of SSS was 31.6% in patients with OSA^72^.

Abe et al.^73^ performed a study on 1,394 Japanese patients and found that CPAP therapy substantially reduced the incidence of AF, PVCs, sinus bradycardia, and sinus pauses.

### OSA and pulmonary hypertension

Pulmonary hypertension (PH) is frequently associated with OSA, and often is a direct consequence of OSA^74^. The prevalence of PH in OSA ranges from 17 to 53%^74^. According the updated guidelines from 6th World Symposium on PH (March, 2019), PH is defined by a mean pulmonary artery pressure (mPAP) >20mmHg rather than >25mmHg, and a pulmonary vascular resistance (PVR) >3 wood units is included to distinguish those with pre-capillary PH^75^.

**Figure 4:**
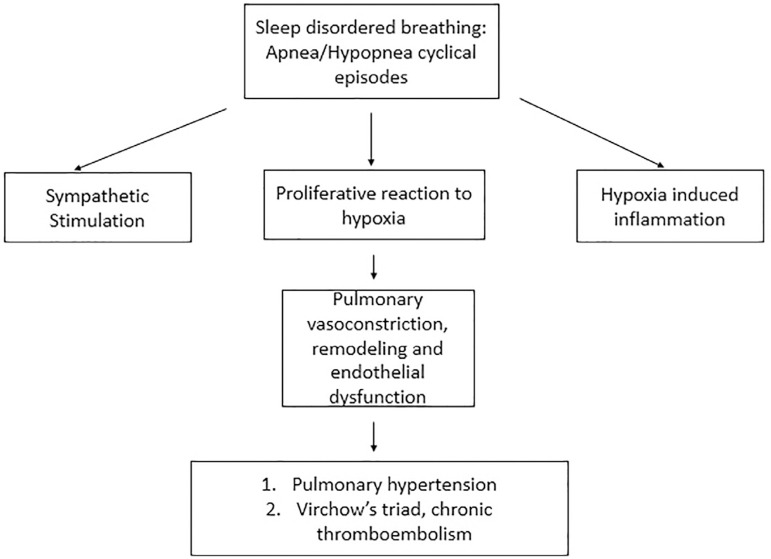
Association of OSA and pulmonary vasculature.

Figure 4 summarizes the three main factors believed responsible for the increase in the PAP observed in sleep apnea:

- Alveolar hypoxia, causing pulmonary vasoconstriction and endothelial remodeling;
- Mechanical related to increased inspiratory effort resulting in, more negative intrathoracic pressure, variations in heart rate and cardiac output, increased left heart filling pressures;
- Reflex mechanisms directly influencing the vasculature^76^.

Acute pulmonary artery pressure changes during sleep have been reported with obstructive apneas^76^. However, the role of OSA as an independent risk factor for the development of daytime PH is not fully established. Severe OSA frequently causes daytime PH in the absence of significant co-existing cardiopulmonary and vascular diseases, and CPAP therapy significantly reduces the levels of daytime pulmonary artery pressure^77^.

Daytime PH may have a precapillary component related to repetitive hypoxia-reoxygenation^78^ leading to both pulmonary vasoconstriction and vascular endothelial remodeling, but there may also be a post capillary component attributable to episodic or permanent elevations in LV filling pressure^79^. The application of nocturnal CPAP therapy can lead to the abolition of both nocturnal hypoxemia and the accompanying sympathetic surges, resulting in improvement in LV diastolic relaxation and decreased LV afterload. This may promote the restoration of the balance among these endothelial vasoactive mediators. Long-term CPAP therapy may avoid irreversible structural pulmonary vascular and right ventricle changes by reducing the pulmonary artery systolic pressure, possibly improving the prognosis^77^.

### OSA and CTEPH

OSA promotes the release of inflammatory markers^56^ and activates the coagulation cascade, which increase the risk of acute thromboembolic events, and also predisposes patients to the development of Type 4 CTEPH (chronic thromboembolic pulmonary hypertension)^80^.

Evidence supports the pathophysiological association of sleep-disordered breathing as an independent risk factor for venous thromboembolism. There is increased prevalence of sleep apnea in patients with acute pulmonary embolism and/or deep vein thrombosis^80^^,^^81^.

Obstructive sleep apnea-related hemodynamic alterations may result in venous stasis, increased thrombogenicity (on a vascular and molecular level), increased inflammatory insult and injury, thus fulfilling the criteria for the nomenclature of Virchow’s triad^82^.

The development of CTEPH may be promoted by the persistence of thrombotic material in the circulation, stemming from inadequate/incomplete thrombus resolution^82^^,^^83^. Chronic hypoxia and hypercapnia in OSA impair thrombus resolution due to inadequate fibrinolysis, persistent inflammation, vascular smooth muscle activation, accelerated adhesion molecule expression and platelet activation^82^^-^^84^.

### OSA and cerebrovascular accidents

Obstructive sleep apnea has been associated with hypoxic-ischemic brain injury (HI-BI), the severity of which depends on the duration and intensity of hypoxemia and ischemia^85^. EEG microarousals and awakenings frequently follow respiratory compromise. Repeated oxyhemoglobin desaturation causes alterations in sympathetic nerve activity, oxidative stress, inflammatory markers and endothelial function, which are associated with decreased vasoreactivity, increased arterial wall stiffness, increased platelet activation and vascular adhesion, resulting in increased risk for cardiovascular and cerebrovascular insults.

Numerous studies have shown the association between OSA and stroke^86^^,^^87^. A systematic review of 37 studies with 3,242 patients showed a high prevalence of OSA in patients with cerebrovascular disease (61.9%)^88^. A meta-analysis of 16 cohort studies reporting data on 24,308 patients demonstrated that moderate and severe OSA are associated with an increased risk of vascular outcomes, including stroke^11^. Patients with OSA are more likely to have a stroke or die than those without OSA^87^. There is an association between OSA and nocturnal cerebrovascular events^89^^-^^91^, and a dose-effect relationship has been described between the adjusted risk and OSA severity, as measured by AHI and oxygen desaturation index, viz., the mean number of desaturations of 4% or more per hour of sleep^86^. OSA contributes to ischemic stroke both as a predisposing risk factor and as a triggering factor; there is a statistically significant association between preceding OSA symptoms and wake-up stroke (WUS)^92^. A higher percentage of cerebral white matter disease, radiographic deep grey matter disease or macroangiopathic strokes is noted in individuals with OSA^89^. A study of 61 patients with silent cerebral infarct and 122 without silent lacunar cerebral infarct demonstrated that the presence of severe OSA syndrome was significantly higher in silent cerebral infarct in comparison to patients without lacunar infarcts (55.8% versus 35.7%, *p*=0.019)^93^. Fluctuations in cerebral blood flow velocity (CBFV) have been documented in OSA, with CBFV increasing along with arterial pressures during OSA episodes. Both CBFV and systemic arterial pressures decrease upon termination of the apneic episode, at the lowest level of oxyhemoglobin desaturation^94^.

Transcranial Doppler imaging has shown decreased cerebrovascular reactivity and increased arterial stiffness, particularly during OSA episodes^95^. An impairment in cerebral autoregulation by means of measurement of the recovery of CBFV and cerebrovascular conductance (CBFV/mean arterial pressure) has been observed after orthostatic challenges, with slower recovery noted in patients with OSA compared to control subjects^96^. A dose-relationship between severity of sleep respiratory disturbance (as measured by AHI) and impaired cerebral autoregulation has also been noted^97^. An impairment in cerebrovascular CO_2_ reactivity, measured by the CBF with increasing AHI was demonstrated in one case control study^98^. Global cerebral blood flow increases during episodes of hypoxemia. A study comparing the increase in CBF in patients with OSA compared to that in healthy subjects demonstrated less increase CBF in OSA patients compared to the control subjects, and this difference was not demonstrable after 3 months of treatment with CPAP^99^. Data from other studies suggest that it is possible to normalize cerebral vasoreactivity and cerebral blood flow with CPAP treatment^100^^,^^101^.

Functional outcomes and long-term mortality of stroke patients with OSA are poor compared to those without OSA. Patients with a higher AHI required longer inpatient rehabilitation and had lower Functional Independence Measure (FIM) scores^102^. Increased mortality 60 months after stroke was observed in those with higher AHI^89^. The nocturnal nadir of oxyhemoglobin saturation is an independent predictor of poor functional outcomes^103^. One study demonstrated that higher severity of SDB correlated with a poorer functional outcome based on the modified Rankin scale score^104^.

There have been concerns about the safety CPAP treatment in patients of acute stroke occurring in the setting of OSA. It is thought that CPAP treatment may reduce cerebral perfusion by altering blood oxygen and carbon dioxide balance. Despite these concerns, current data obtained from prospective and cohort studies suggest no adverse effect of CPAP treatment in patients with OSA during acute stroke^89^^,^^105^.

The effect of continuous positive airway pressure (CPAP) treatment was evaluated as a primary outcome measure for prevention of new vascular events among OSA patients with stroke. Concurrently, secondary outcome measures were designed to assess post stroke clinical outcomes utilizing the Barthel index and the modified Rankin scale. Measurement of neuropsychological parameters suggested better stroke outcomes and there was a trend toward favorable outcomes vis-a-vis reduced recurrence of vascular events^35^. A meta-analysis of seven randomized controlled trials which included 4,268 patients showed a significant reduction in relative risk or major adverse cardiovascular events and stroke, which correlated with increased CPAP usage time (adherence time >4 hours)^106^. Another systematic review and meta-analysis of 4 randomized clinical trials and 1 prospectively matched observational cohort, (total of 389 patients) showed a mean decrease in National Institutes of Health Stroke Scale scores during the first (≤30) days of acute ischemic stroke in patients treated with non-invasive ventilation (NIV) compared to control subjects (standardized mean difference, 0.38; 95% confidence interval, 0.11-0.66; *p*=0.007)^107^.

Although several studies have demonstrated the beneficial effect of CPAP on recovery outcomes in stroke patients, including more rapid functional recovery, reduced hospitalization time, and decreased frequency of re-hospitalization, significant challenges remain due to post stroke disability which may lead to limited CPAP adherence in the hospital environment.

Consequently, there has been increased interest in alternate options to treat SDB such as mandibular advancement surgery and supine avoidance. Additional studies are needed to evaluate their efficacy in post-stroke rehabilitation outcomes^108^.

### OSA and other neurological disorders

OSA increased the risk of developing optic neuropathy after controlling for comorbidities as demonstrated in a Taiwanese population-based cohort study; however, treatment with CPAP did not reduce the risk of optic neuropathy^109^.

Review performed by Chaitanya et al.^110^ highlights OSA as a risk factor for developing glaucoma. Another important point to note is that CPAP therapy can trigger glaucoma damage by raising the intraocular pressure (IOP), which would warrant glaucoma screening in patients on CPAP.

A study to investigate the association between obstructive sleep apnea (OSA) and middle ear acoustic transference/cochlear function demonstrated that severe OSA is associated with cochlear function impairment in patients. Patients with severe OSA presented with significantly lower distortion product otoacoustic emissions (DPOAE) amplitudes when compared to the control, mild, and moderate OSA groups^111^.

Fecal and urinary incontinence has been reported to resolve after treatment with positive airway pressure non-invasive ventilation^112^ in a patient with obstructive sleep apnea hypopnea syndrome (OSAHS). Five adults OSA patients who presented with enuresis, enuresis resolved after treatment with continuous positive airway pressure (CPAP)^113^, and similar improvement was noted in another case report^114^.

Patients with OSA demonstrate impairments in behavior, cognition, and physical skills^85^. Excessive daytime sleepiness, as measured by the subjective Epworth sleepiness scale (ESS) and the objective multiple sleep latency test (MSLT) and the maintenance of wakefulness test (MWT) is the most common neurobehavioral consequence of OSA. Other behavioral problems occurring in this patient population include disinhibition, distractibility, and irritability^115^^,^^116^. Physical skills and cognitive abilities, including selective attention, vigilance, short-term and working memory, and executive and motor functioning are adversely affected by OSA^115^^-^^121^. Most neurobehavioral deficits, except executive dysfunction, have been found to be reversible with CPAP treatment of OSA^118^^,^^122^^-^^124^. CPAP treatment for as little as 2 weeks has been shown to improve daytime sleepiness, including reduction in subjective sleepiness as measured by the ESS, but no significant changes were observed in objective sleepiness measured by MSLT or MWT^125^^-^^127^. Several key neurobehavioral indices (functional outcomes of sleep questionnaire, Epworth sleepiness scale) failed to normalize despite 3 months of CPAP treatment, even in those who were maximally compliant with treatment. Forty percent of patients in this trial had an abnormal Epworth sleepiness scale score at the conclusion of the trial^128^. Despite this, CPAP treatments have been shown to result in significant improvement in attention, alertness, speed of visual motion perception, vigilance, speed of information processing immediate visual memory, working memory and cognition^122^^,^^126^^,^^129^. A prospective 12-month observational study of CPAP treatment of OSA assessed its effects on non-motor symptoms in 67 patients with Parkinson’s disease. Overall improvement in non-motor symptoms, sleep quality, anxiety, and global cognitive function were observed^130^.

### Diagnosis

Clinical symptoms play a key role in the diagnosis of OSA, although no sign or symptom is specific for the diagnosis of OSA. Once there is a high suspicion questionnaires and symptom- scoring scales can be used to increase the accuracy of diagnosis. Screening questionnaires are used in the outpatient setting, for symptomatic patients, to determine whether a patient should undergo polysomnography. Polysomnography is the standard for diagnostic confirmation, however it is expensive and not always available^2^.

The Mallampati classification (examination of the oropharyngeal inlet) is used to evaluate if tonsillar, uvular, and tongue enlargement are affecting the airway volume^8^.

### Pathophysiology

Activation of the pro-inflammatory transcription factor nuclear kappa factor B (NF-kB) by apnea-induced hypoxia is an important pathway linking obstructive sleep apnea with systemic inflammation. It can also stimulate the downstream inflammatory markers resulting in end- organ cardiovascular disease. NF-kB activity is elevated in circulating neutrophils and monocytes in patients with obstructive sleep apnea and studies have revealed decreased activity with continuous positive airway pressure therapy in adults^131^^-^^133^.

### Questionnaires

Questionnaires are sensitive however not very specific, therefore when a patient has low scores, it is helpful to attempt to reduce the diagnostic likelihood of OSA, in some instances avoiding the need to proceed with polysomnography^134^^,^^135^.

#### STOP-bang

The STOP-bang questionnaire includes questions on snoring, tiredness, observed apneas, blood pressure, BMI, age, neck circumference, and gender. It is one of the most sensitive questionnaires available for use in the clinical setting^135^. Every parameter is scored one point; and a score of >3 indicates a high risk of OSA.

Sleep apnea clinical score (SACS)

It includes data on the neck circumference, hypertension, habitual snoring, and nocturnal gasping or choking. The score ranges from zero to 100 and a score greater than 15 increases the likelihood of being positive for OSA.

#### Berlin questionnaire

The questionnaire has ten sections distributed in three categories, which include data on snoring, non-restorative sleep, sleepiness while driving, apneas during sleep, hypertension, and BMI. Points are assigned for each category and the patient is identified as high risk or low risk based on the points^135^.

### NoSAS

The system assesses five components including neck circumference, BMI, snoring, age, and sex. A cut-off of eight points is used to identify patients with sleep-disordered breathing^136^.

### Polysomnography (PSG)

PSG is the standard procedure for the diagnosis of OSA^2^. The preferred approach is to perform overnight PSG in the sleep laboratory; however, it is costly and may not be available (nor approved by third party insurers) at all times. Therefore, home sleep apnea testing (HSAT) can be used for certain patient populations^137^. HSAT can be performed for patients who have a high pre-test probability of OSA and do not have other comorbidities^137^. However, if a HSAT is inconclusive, inadequate or negative, PSG should be performed^137^. Patients with comorbidities such as cardiovascular disease, respiratory muscle weakness secondary to neuromuscular disorders, history of strokes or other ischemic disease, and chronic opioid use should undergo PSG rather than HSAT^137^.

Several parameters are monitored during PSG. Electroencephalography (EEG), chin electromyography (EMG) and electrooculography (EOG) are done to identify episodes of arousal and to determine sleep stage^2^. Respiratory airflow recommended: simultaneous monitoring of two physical variables: air temperature (for thermal airflow) and air pressure (for nasal pressure), respiratory effort, oxyhemoglobin saturation, and ECG are monitored^21^. To diagnose OSA, apnea-hypopnea index (AHI) is measured, i.e., the number of apneic and hypopneic episodes per hour of sleep are tabulated^21^. Apnea is an episode of stoppage of respiratory airflow for a minimum of 10 seconds. Hypopnea is the decrease in airflow, associated with either a drop-in oxyhemoglobin saturation or an episode of arousal^21^. AHI of greater than five is diagnostic of OSA. AHI greater than or equal to five but less than fifteen is classified as mild, greater than or equal to fifteen but less than thirty is classified as moderate, and greater than or equal to thirty is classified as severe OSA^22^.

### Management of OSA

CPAP is the primary management strategy for OSA as it decreases symptoms of sleepiness and improves quality of life in patients with moderate and severe disease^2^^,^^138^. CPAP treatment prevents (or ameliorates) collapse of the upper airways^137^. Change in dietary habits, regular exercise, and weight loss can also contribute to the management of OSA^139^. Bariatric surgery is an option in extreme cases, and may be associated with significant improvement; however, it has not been shown to totally reverse OSA and does not replace the use of CPAP as the primary treatment^140^. However, after surgery and significant weight loss polysomnography should be repeated and CPAP titration should be performed^141^. Other surgical options include tonsillectomy, uvulopalatopharyngoplasty, tongue surgery (to reduce the size) and maxillomandibular advancement surgery^142^. Another alternative is the use of mandibular advancement devices (MAD). The purpose of the device is to expand and stabilize the airway and to lessen the collapse. Although these are not as effective as CPAP in reducing AHI^142^^,^^143^ they can be used in certain circumstances when there is insufficient compliance with CPAP use.

Wojda et al.^144^ performed a clinical study with 8 patients, comparing the use of CPAP and MAD and found that the symptoms improved to greater extent with CPAP. In cases where adherence to CPAP is low, these alternative options can be considered. However, CPAP is treatment of choice and has shown the best outcomes, including reduction in all-cause mortality^2^^,^^138^^,^^145^. Yearly follow up should be performed after CPAP is initially set up^146^. Oral appliances can be used for patients mild to moderate OSA under certain conditions^146^. Ramar et al.^147^ published recommendations based on an extensive review that endorsed the use of oral appliances for patients with snoring (without OSA) and for patients with OSA that are intolerant of CPAP or prefer an alternate treatment. Their guidelines also included the use of custom oral devices for patients with oversight by qualified dentists to monitor for dental side effects, and follow up testing by sleep physicians to check for treatment effectiveness.

## CONCLUSION

OSA affects multiple organ systems. OSA may first present with cardiovascular or neurological morbidity, rather than respiratory symptomatology. It is important to use clinical judgement and to keep a low threshold for diagnosis when patients present with these varied signs and symptoms in order to make a timely diagnosis and to intervene to prevent morbidity and mortality.
